# Roles of Natural Killer T Cells and Natural Killer Cells in Kidney Injury

**DOI:** 10.3390/ijms20102487

**Published:** 2019-05-20

**Authors:** Takahiro Uchida, Seigo Ito, Hiroo Kumagai, Takashi Oda, Hiroyuki Nakashima, Shuhji Seki

**Affiliations:** 1 Department of Nephrology and Endocrinology, National Defense Medical College, 3-2 Namiki, Tokorozawa, Saitama 359-8513, Japan; seigoemon@yahoo.co.jp (S.I.); hkumagai@ndmc.ac.jp (H.K.); 2 Department of Nephrology, Tokyo Medical University Hachioji Medical Center, Hachioji, Tokyo 193-0998, Japan; takashio@tokyo-med.ac.jp; 3 Department of Immunology and Microbiology, National Defense Medical College, Tokorozawa, Saitama 359-8513, Japan; hiro1618@ndmc.ac.jp (H.N.); btraums@ndmc.ac.jp (S.S.)

**Keywords:** acute kidney injury, CD56^+^ T cell, lupus nephritis, natural killer T cell

## Abstract

Mouse natural killer T (NKT) cells and natural killer (NK) cells are innate immune cells that are highly abundant in the liver. In addition to their already-known antitumor and antimicrobial functions, their pathophysiological roles in the kidney have recently become evident. Under normal circumstances, the proportion of activated NKT cells in the kidney increases with age. Administration of a synthetic sphingoglycolipid ligand (alpha-galactosylceramide) further activates NKT cells, resulting in injury to renal vascular endothelial cells via the perforin-mediated pathway and tubular epithelial cells via the TNF-α/Fas ligand pathway, causing acute kidney injury (AKI) with hematuria. Activation of NKT cells by common bacterial DNA (CpG-ODN) also causes AKI. In addition, NKT cells together with B cells play significant roles in experimental lupus nephritis in NZB/NZW F1 mice through their Th2 immune responses. Mouse NK cells are also assumed to be involved in various renal diseases, and there may be complementary roles shared between NKT and NK cells. Human CD56^+^ T cells, a functional counterpart of mouse NKT cells, also damage renal cells through a mechanism similar to that of mice. A subpopulation of human CD56^+^ NK cells also exert strong cytotoxicity against renal cells and contribute to the progression of renal fibrosis.

## 1. Introduction

Mouse natural killer T (NKT) cells, which express both NK1.1 antigen and the intermediate T-cell receptor (TCR), as well as NK cells, are innate immune cells that are present in abundance in the liver. When these cells are activated by various stimuli, including cytokines and bacterial components, they play crucial roles in the defense against tumors and bacterial infections via the IFN-γ/perforin pathway [1]. Alpha-galactosylceramide (α-GalCer) is a synthetic sphingoglycolipid ligand of NKT cells [2,3], which also activates NKT cells and induces antitumor responses that are mediated by NK cells and subsequently CD8^+^ T cells [4]. However, if NKT cells are inadequately activated, septic shock or multiple organ failure via TNF-α/Fas ligand (FasL) may occur [5].

In humans, T cells that express TCRs encoded by the *Vα24Jα18* and *Vβ11* genes, which have an arrangement resembling that of mouse invariant NKT cells, were suggested to be NKT cells [6]. In fact, these cells are activated by α-GalCer; however, they exist only in small numbers both in the peripheral blood and liver [7]. On the other hand, human CD56^+^ T cells are considered to be a functional counterpart of mouse NKT cells, because (i) they express a surface marker of NK cells (CD56) and intermediate and oligoclonal TCRs [1,8,9]; (ii) they are present abundantly in the liver; (iii) they exert antitumor cytotoxicity after cytokine stimulation and are thought to be involved in the inhibition of hepatocellular carcinoma development [7]; and (iv) most (approximately three quarters) of liver CD56^+^ T cells also express CD161, a NK cell receptor protein 1 (NKR-P1) molecule to which the NK1.1 antigen in mice belongs [7,10,11]. Therefore, in this review we consider human NKT cells to be cells that express αβTCR and CD56 (CD56^+^ T cells), unless otherwise specified. However, it should be noted that whereas mouse NKT cells are almost exclusively either CD4^+^ or CD4^−^ CD8^−^ [12], human CD56^+^ T cells are regularly CD8 [9,10].

In addition to the already-known antitumor or antimicrobial functions, the involvement of the above cells in various renal diseases has recently been investigated in detail. In this review, we will give an overview and discuss the recent advances in the understanding of the roles of NKT and NK cells in kidney injury both in mice and in humans.

## 2. Mouse Natural Killer T (NKT) Cells and Natural Killer (NK) Cells in the Kidney under Normal and Activated Conditions

As with the liver, the normal kidney contains innate immune lymphocytes, including NKT and NK cells; both the proportion of NKT cells and that of NK cells in the kidney are higher than that of the spleen and blood [13]. This may suggest that the kidneys play important roles in the innate immune response. Although the proportion of NKT cells in the kidney remains unchanged with age, the proportion of NKT cells expressing CD69, a marker of their activation, increases with age [14]. The proportion of activated NKT cells in the kidneys also increases in mice depleted of NK cells by an anti-asialo-GM_1_ antibody. IL-12 administration increases the proportion of NKT cells in the kidneys, consistent with previous reports showing that NKT cells activated by IL-12 migrate from the liver and suppress renal metastasis of malignant tumors [1,9].

## 3. Functions and Roles of Mouse NKT Cells in Renal Diseases and Pathological Conditions

Previous studies have suggested the regulatory roles of mouse NKT cells in various renal diseases [15]; however, their roles appear to be more complicated than previously considered. We herein describe in detail how NKT cells are associated with renal diseases, including in kidney transplantation rejection.

### 3.1. Acute Kidney Injury (AKI)

**Figure 1:**
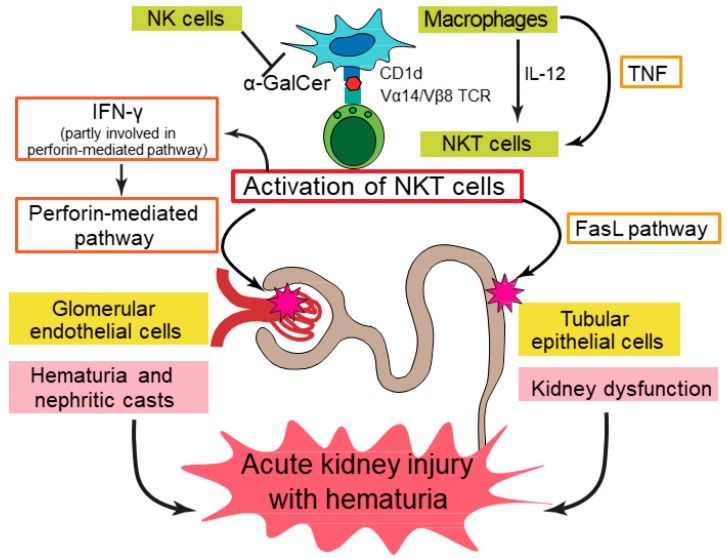
Putative pathogenic mechanisms underlying kidney injury induced by alpha-galactosylceramide (α-GalCer)-stimulated natural killer T (NKT) cells in mice. Activated NKT cells cause hematuria and nephritic casts by damaging glomerular endothelial cells in a perforin-dependent manner, whereas they damage tubular epithelial cells via Fas ligand (FasL) pathway activation, which leads to kidney dysfunction. IFN-γ produced by activated NKT cells is partly involved in perforin pathway activation, and TNF-α produced by macrophages is mainly involved in activation of the FasL pathway. Both pathways exert independent effects, thereby inducing acute kidney injury with hematuria. IL-12 enhances α-GalCer-activated functions of NKT cells, whereas NK cells may play a protective role in this model. This scheme is based on the figure from Uchida et al. [14].

Although α-GalCer has been shown to activate NKT cells and cause the failure of multiple organs, including the liver, lung, and kidney (AKI), particularly in aged animals [5], the precise mechanisms of this AKI remain unclear. We have recently shown that α-GalCer activates NKT cells in the kidney, thereby injuring both renal vascular endothelial cells and tubular epithelial cells, and causing AKI with hematuria both in C57BL/6J (B6) [14] and BALB/c mice. Acute tubular injury was pathologically confirmed to occur in α-GalCer-injected mice. Interestingly, the perforin-mediated pathway and TNF-α/FasL system independently play important roles in this model; treatment with concanamycin A, a perforin blocker, significantly decreased the cytotoxicity of α-GalCer-activated mononuclear cells (MNCs) against renal vascular endothelial cells, whereas the inhibition of TNF-α or FasL significantly decreased the cytotoxicity against tubular epithelial cells, suggesting that the former is exclusively involved in vascular endothelial cell injury and the latter mainly affects the injury of tubular epithelial cells. In addition, the function of NKT cells in this model was enhanced in mice depleted of NK cells by receiving anti-asialo-GM_1_ antibody injections (Figure 1, based on Uchida et al. [14]). 

Although definitive natural ligands of NKT cells have not been identified so far, some microbes have been shown to be their antigenic targets [16,17]. In addition, not only α-GalCer, but also CpG-ODN, bacterial DNA motifs, activate NKT cells and cause renal damage accompanied by acute tubular injury, and the involvement of TNF-α/FasL pathway is suggested [18]. It is, therefore, strongly suggested that NKT cells play important roles in the disease progression of AKI in response to various microbes. Moreover, multiple organ failure (liver, lung as well as kidney) was found to occur in the generalized Shwartzman reaction, which is induced by IL-12 priming of NKT cells as well as CD8^+^ CD122^+^ T cells to produce IFN-γ, together with the subsequent lipopolysaccharide challenge that induces TNF-α production [19,20]. Furthermore, vascular endothelial cell injury in this model occurs in a perforin-dependent but FasL pathway-independent manner [21]. Taken together, the perforin-mediated pathway and TNF-α/FasL system may interact in a complicated manner in the pathophysiology of the disease.

In contrast to conventional T cells and B cells, NKT cells have been reported to be resistant to treatment with glucocorticoids as well as irradiation [22]. We also observed that kidney and liver injury after α-GalCer injection tended to be increased in B6 mice pre-exposed to 4 Gy of total body irradiation as compared with that in untreated mice (Table 1), although 4 Gy of irradiation alone did not induce any liver injury (the level of alanine aminotransferase did not increase at all),. Of particular interest, serum IFN-γ levels after α-GalCer injection were significantly higher in mice receiving 4 Gy of irradiation, suggesting that the function of NKT cells was rather enhanced by irradiation in these mice. In addition, the proportion of NKT cells among total liver MNCs was also increased by 4 Gy of irradiation, but the proportions of conventional T cells and B cells decreased. Furthermore, the levels of IFN-γ and IL-4 produced by liver MNCs following α-GalCer stimulation in B6 mice pre-exposed to 4 Gy of irradiation tended to be increased compared with those in B6 mice without irradiation. Therefore, when considering treatment options for AKI induced by NKT cells, strategies other than irradiation or glucocorticoids to inhibit perforin or TNF-α/FasL seem to be reasonable and should be carefully investigated in the future. 

The pathogenic roles of NKT cells have also been demonstrated in renal ischemia-reperfusion injury (IRI) models, which are representative experimental models of AKI. One report showed that the absence of NKT cells markedly attenuated renal damage, including tubular necrosis, suggesting the role of NKT cells in the pathogenesis [23]. More recently, it was reported that IL-33, together with IL-12, promoted the recruitment of NKT cells and the production of cytokines such as IFN-γ and IL-17 by these cells, thereby inducing the IRI [24]. In addition, involvement of the FasL pathway in mediating renal tubular injury in that model has been suggested [25]. In contrast, the renoprotective functions of NKT cells have also been reported in another study [26]. In this regard, differences in the phase or severity of renal injury might affect NKT cell function. It should also be stressed that NKT cells may participate in tissue restoration/regeneration and homeostasis; they have been shown to accelerate liver regeneration after hepatectomy using the TNF-α/FasL system [27]. NKT cells may therefore induce both renal tubular damage and its repair via the same apoptotic process, namely the TNF-α/FasL pathway.

### 3.2. Lupus Nephritis

Systemic lupus erythematosus (SLE) is a representative systemic autoimmune disease, which is characterized by the presence of autoantibodies and involves virtually any organ. Lupus nephritis is a serious complication involving almost half of all SLE patients, which results in an unfavorable renal prognosis [28,29]. The roles of NKT cells in the pathogenesis of SLE is controversial. Analysis of a mouse model of SLE indicated that the expansion of NKT cells is involved in the onset of lupus nephritis (Table 2) [30], whereas their immunoregulatory roles have also been reported [31,32,33].

The effects of α-GalCer in the progression of SLE models have also remained contradictory (Table 2). In MRL/lpr mice, which is an SLE model with a defective point mutation in Fas, α-GalCer treatment resulted in an improvement in inflammatory dermatitis without affecting renal disease and enhanced levels of anti-dsDNA antibodies [34]. Repeated α-GalCer treatment suppressed pristane-induced lupus nephritis in BALB/c mice but exacerbated the disease in SJL mice [35]. 

NZB/NZW F1 (BWF1) mice, which is another representative SLE model, spontaneously produce autoantibodies and develop lupus nephritis-like renal lesions [36], and therefore are considered to resemble the human disease. Long-term administration of a neutralizing anti-NK1.1 antibody into these mice ameliorated lupus nephritis in the late disease phase while worsening it in the early phase (Table 2) [37]. On the other hand, previous studies have reported contradictory results regarding the effects of α-GalCer in BWF1 mice (Table 2). Thus, Zeng et al. reported that multiple injections of α-GalCer to adult BWF1 mice up-regulated the functions of NKT cells and exacerbated lupus nephritis by enhancing Th1 immune responses [38]. On the contrary, Yang et al. demonstrated that brief treatment with α-GalCer of young BWF1 mice reduced IL-10 production and induced the long-term reduction of severe proteinuria in these mice [39]. The complex role of NKT cells in SLE, i.e., a potential protective role before disease onset and a potential pathogenic role after disease establishment, might have caused the contradictory results; however, the molecular mechanisms underlying these differences remain unclear. Based on the above, we investigated the involvement of NKT cells in lupus nephritis using adult BWF1 mice. We clearly showed that the repeated administration of α-GalCer into these mice not only induced an anergic state to α-GalCer in NKT cells, as previously described [40], but also decreased the number of NKT cells in multiple organs and suppressed Th2 immune responses in these cells without affecting their Th1 immune responses, leading to the suppression of B-cell function and amelioration of experimental lupus nephritis (Table 2) [41]. The different effects of α-GalCer may have led to the conflicting results between the study by Zeng et al. [38] and our study [41]. Regarding this point, the dose of α-GalCer, its administration interval, or age of the mice used might have affected the results. NKT cells have been reported to cooperate with B cells to generate immunoglobulins, including autoantibodies, and the involvement of both cellular interaction (via CD1d and CD40/CD40L molecules) and cytokine secretion (IL-21 produced by NKT cells) has been suggested [42,43]. Whether this interaction takes place in the kidney or not remains unclear because it has been mainly investigated in an in vitro study using spleen cells. However, it should also be taken into consideration that α-GalCer injections have been reported to modulate immune responses towards a Th2 phenotype in normal mice [44] and that Th2-biased immune responses induced by the administration of α-GalCer [45,46] or its derivative [47] prevents diabetes in nonobese diabetic mice. The effects of α-GalCer may therefore vary depending on the disease models and the mouse strains.

### 3.3. Other Renal Diseases

In adriamycin-induced nephropathy, a model of focal segmental glomerulosclerosis in which chronic proteinuric renal injury is shown, an agonist of NKT cells was reported to modulate immune responses and inhibit the development of the disease model [48]. There have also been several studies showing the protective roles of NKT cells in some glomerulonephritis or vasculitis models, and the production of cytokines, such as IL-4, IL-10, and TGF-β was suggested to play various immunoregulatory effects [49,50,51]. The absence of NKT cells exacerbated an experimental model of tubulointerstitial nephritis [52]. In this model, α-GalCer treatment reduced renal injury, suggesting that α-GalCer-induced IFN-γ production contributes to the improvement of renal injury. Thus, NKT cells may play complex roles by exerting different immunoregulatory functions in various renal diseases.

### 3.4. Renal Transplantation

The role of NKT cells in the field of organ transplantation is controversial; they are assumed to be involved in immune tolerance after liver transplantation [53], whereas they have been reported to play a role in the rejection of islet allografts in the liver [54]. There is a very small amount of data to our knowledge regarding their involvement in renal transplantation to date.

## 4. Role of Mouse NK Cells in Kidney Injury

Similar to NKT cells, the frequency of NK cells as well as their activation in the kidney have been reported to be increased by renal IRI, suggesting their deleterious roles. The up-regulated expression of a NK cell ligand on tubular epithelial cells and its engagement through the activating receptor NKG2D is reportedly involved in the pathogenesis of renal IRI [55]. The involvement of NK cells in the generalized Shwartzman reaction through damage to vascular endothelial cells, presumably via the perforin-mediated pathway, has also been reported [21].

Although some studies suggested that NK cells in the kidney are activated and that they may be responsible for promoting and maintaining inflammation in lupus nephritis [56,57], data with respect to the role of NK cells in experimental lupus nephritis is limited. As described above, NK cells were activated in BWF1 mice in which repeated α-GalCer administration induced anergy in NKT cells. On the contrary, in the α-GalCer-induced AKI model, the function of NKT cells was augmented in the absence of NK cells. Therefore, although the precise mechanism remains to be solved in future studies, there may be complementary roles shared between NKT and NK cells to avoid immunosuppressive states. In contrast to the reported immunomodulatory roles of NKT cells, NK cells may not play significant roles in adriamycin-induced nephropathy [58].

NK cells have been reported to mediate transplanted kidney injury [59]. However, it should be noted that antibody-mediated rejection (ABMR) models of kidney transplantation have not been well established yet [60].

## 5. CD56^+^ T Cells Act as Human NKT Cells in Kidney Injury

Human CD56^+^ T cells, as well as (CD56^+^) NK cells, have been shown to play significant roles in generalized Shwartzman reaction-like responses in vitro [61]. Consistently, our recent research has shown that CD56^+^ T cells stimulated by a combination of IL-2 and IL-12 demonstrates significantly stronger cytotoxicity against both glomerular endothelial cells and tubular epithelial cells than regular T cells [14]. The cytotoxicity against glomerular endothelial cells was significantly decreased by inhibition of the perforin-mediated pathway. CD56^+^ T cells have been shown to produce large amounts of IFN-γ, perforin, and soluble FasL [8,62]. CD56^+^ T cells therefore potentially damage intrinsic renal cells and can be integral to the processes that mediate AKI. In addition, there may be a common pathogenesis between the conditions in mouse NKT cells and human CD56^+^ T cells. Recently, it has been reported that γδT cells with NK cell–associated markers (including CD56^+^ T cells of the γδ type) are associated with renal fibrosis [63]. Our investigation showed that after stimulation with IL-2, IL-12, and IL-15, γδT cells, both with and without CD56 expression, exerted strong cytotoxicity against renal tubular epithelial cells, supporting a previous study [64] and further clarifying the pathogenesis of these cells.

As described above, mouse NKT cells may cooperate with B cells to play significant roles in experimental lupus nephritis through their Th2 immune responses. In this regard, it should be noted that CD56^+^ T cells do not produce very much IL-4, and hence the condition in mice may be different to that in humans. Indeed, a decreased number of human CD56^+^ T cells was reported to be associated with high levels of serum IgG and anti-dsDNA antibodies in patients with SLE [31], suggesting that CD56^+^ T cells may ameliorate human SLE. Whether human NKT cells are actually involved in the pathogenesis of lupus nephritis should therefore be carefully investigated.

Whether CD56^+^ T cells are involved in other human renal diseases has not been well investigated, and data showing their involvement are essentially limited to case reports and case series. In patients with Balkan endemic nephropathy, which is a chronic tubulointerstitial renal disease that is associated with increased incidence of upper urinary tract urothelial carcinoma, the number of CD56^+^ T cells in peripheral blood is reportedly increased during disease progression, suggesting that these cells are associated with disease pathogenesis [65]. Case reports have also suggested that NK/T-cell lymphoma is associated with active glomerulonephritis, such as crescentic glomerulonephritis [66] or IgA nephropathy [67].

One study reported that CD56^+^ T cells participate in tubular necrosis or the rejection of transplanted kidneys [68]. Another study demonstrated that a large number of CD56^+^ T cells in the peripheral blood were associated with an unfavorable outcome of renal grafts [69].

## 6. Human CD56^+^ NK Cells in Kidney Injury

We have shown that when stimulated by a combination of IL-2 and IL-12, CD56^+^ NK cells exert strong cytotoxicity against intrinsic renal cells [14]. As described above [61], these cells may also induce tissue damage, leading to multiple organ failure. However, under normal conditions, NK cells receive inhibitory signals from cells expressing major histocompatibility complex (MHC) class I molecules, and do not damage them. Then, how do they target these “normal” cells?

**Figure 2:**
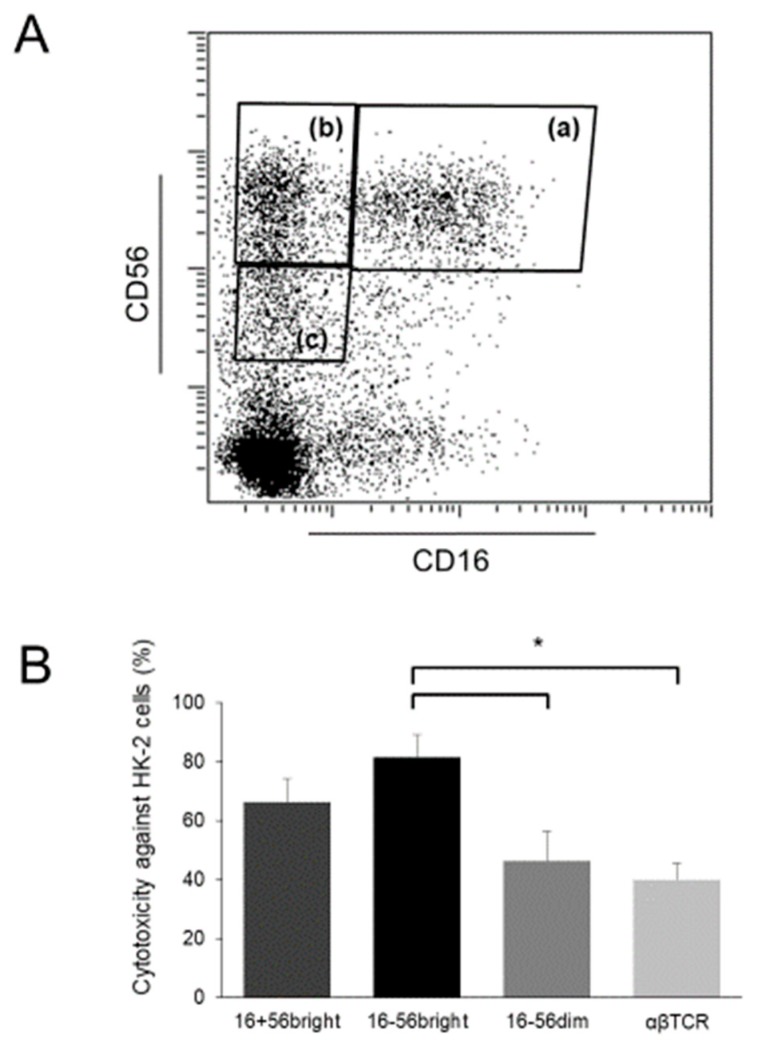
Proliferation of CD56^bright^ cells with strong cytotoxicity against renal tubular epithelial cells after cytokine stimulation. (**A**) Flow cytometric profiles of NK cell subsets. Human peripheral blood mononuclear cells were isolated and cultured for 14 days under stimulation with IL-2, IL-12, and IL-15. Thereafter, they were sorted into CD16^+^ CD56^bright^ cells (a), CD16^−^ CD56^bright^ cells (b), CD16^−^ CD56^dim^ cells (c), and αβTCR^+^ cells using a cell sorter; (**B**) Cytotoxic activity of cells analyzed using the calcein-acetyoxymethyl cytotoxicity assay. The sorted cells in (**A**) were cultured again with the same procedure overnight to recover their function from the damage by sorting. These cells and human proximal tubular epithelial HK-2 cells (ATCC, Manassas, VA, USA) were used as effector cells and target cells, respectively. Effector-to-target ratios were 5:1. Data are presented as arbitrary units ± standard of error of the mean (*n* = 3 in each group); * *p* < 0.05.

Whereas most NK cells among peripheral blood MNCs are CD16^+^ CD56^dim^ cells, CD16^−^ CD56^+^ cells are the major NK cells in tissues, including the liver, and there is a discrepancy between NK cells in the peripheral blood and those in tissues [9]. CD16^−^ CD56^+^ cells in peripheral blood are not cytotoxic under normal circumstances; however, when activated by several cytokines, some of them acquire CD16 expression and show up-regulated CD56 expression, and these cells produce large amounts of IFN-γ and exert strong antitumor cytotoxicity against not only MHC class I-negative but also MHC class I-positive (that is, NK-resistant) tumor cells [70]. In fact, it has been reported that cytokine-stimulated CD56^bright^ NK cells show significantly stronger cytotoxicity than CD56^dim^ NK cells (and T cells) against human umbilical vein endothelial cells [1]. In patients with Kawasaki disease, which is a primary systemic vasculitis with predominant medium-sized vessel involvement [71], affected patients have pyuria with a large number of CD56^bright^ NK cells in their urine [1], suggesting the involvement of these cells in the injury of vascular endothelial cells. Our recent investigation using cytokine-stimulated lymphocytes showed that CD56^bright^ cells, both with and without CD16 expression, strongly injure renal tubular epithelial cells. In particular, the cytotoxicity of CD16^−^ CD56^bright^ NK cells was significantly higher than that of CD56^dim^ cells and T cells (Figure 2).

Although studies analyzing renal NK cells are limited, it has been suggested that normal kidneys as well as kidneys from patients with different forms of renal diseases contain a substantial number of CD56^+^ (including CD56^bright^) NK cells other than CD56^dim^ cells [72]. In contrast to CD56^+^ cells in peripheral blood, these CD56^+^ cells in the kidney may cause pathogenic effects. There are significantly more CD56^+^ cells among urinary MNCs than peripheral blood MNCs in patients with IgA nephropathy, which is a representative type of chronic glomerulonephritis [73]. CD56^+^ (and CD16^+^) NK cells have also been suggested to induce hematuria in IgA nephropathy [74]. In addition, several reports in support of the data that NK cells are involved in the pathogenesis of IgA nephropathy have been published [75,76]. CD56^bright^ NK cells were also reportedly associated with the degree of fibrosis and loss of renal function and had increased expression of the activation marker CD69 and the activated NK cell receptor NKp46 [72]. These cells were localized to sites of tubulointerstitial injury and they expressed IFN-γ, suggesting that NK cells, particularly CD56^bright^ NK cells, play important roles in the disease progression of renal fibrosis [72].

A counterpart of human CD56^bright^ NK cells has not yet been identified in mice, because mouse NK cells do not express CD56, making it difficult to further understand the biology of human CD56^bright^ NK cells, such as their functions, activation mechanisms, and factors affecting their functions. However, activated NK cells in mice also acquire cytotoxic activity against various tumors, including NK-resistant tumors [4], suggesting the possibility that NK cells with a similar function exist in mice.

Both numbers of NK cells in peripheral blood and their antitumor cytotoxicity are reportedly decreased in renal transplant recipients treated with standard immunosuppressive therapy, although the number of CD56^+^ T cells remained unchanged after renal transplantation [12]. However, NK cells are considered to be deeply involved in ABMR of transplanted kidneys [60]. They presumably bind to the endothelium of the microcirculation through CD16a (Fc gamma receptor IIIA)-mediated recognition of the Fc portion of anti-donor antibodies and are activated, thereby producing cytokines and exerting cytotoxicity. Activating signals via complement receptors on NK cells and NKG2D, their activating receptor, may also be involved in their pathogenic roles [77].

NKG2D expression in NK cells has been reported to be decreased in patients with end-stage renal disease who are receiving dialysis therapy [78]. This decrease may be associated with impaired immune functions in these patients, leading to a significant increase in the incidence of infections or malignancies.

## 7. Concluding Remarks

In recent years, it has become evident that NKT and NK cells play significant roles in various renal diseases, including acute kidney injury and immune-mediated glomerulonephritis, as well as in the field of renal transplantation. In this review, we provided an overview and discussed recent advances in our understanding of the roles of NKT and NK cells in kidney injury, both in mice and humans.

Activated mouse NKT cells cause AKI with hematuria through damage to renal vascular endothelial cells via the perforin-mediated pathway, and to tubular epithelial cells via the TNF-α/Fas ligand pathway. Human CD56^+^ T cells, a functional counterpart of mouse NKT cells, also cause damage to intrinsic renal cells, and there may be a common pathogenic pathway between mouse and human conditions. Repeated administration of α-GalCer slows the progression of experimental lupus nephritis in BWF1 mice by decreasing the number of NKT cells and suppressing Th2 immune responses in these cells as well as by decreasing B-cell function. However, whether human NKT cells are involved in the pathogenesis of lupus nephritis should be further investigated, because these cells may not induce Th2 immune responses. Mouse NK cells may also be involved in various renal diseases, including AKI. In addition, there may be interesting roles shared between NKT and NK cells, which function to avoid immunosuppressive states; the function of NKT cells is up-regulated in the absence of NK cells, whereas NK cells are under activation when NKT cells are in an anergic state. A subpopulation of human CD56^+^ NK cells, namely CD56^bright^ cells, cause substantial damage to intrinsic renal cells, and these cells may also be involved in the pathogenesis of fibrotic kidney as well as immune-mediated glomerulonephritis. Data showing the involvement of NKT and NK cells in the injury of transplanted kidneys have been increasing, and future studies in the field of renal transplantation, which focuses on these innate immune cells are anticipated.

## Figures and Tables

**Table 1:** Effects of 4 Gy of total body irradiation on organ damage and cytokine levels after alpha-galactosylceramide injection.

	Irradiation (−)	Irradiation (+)
Number of mice	3	3
Blood urea nitrogen (mg/dL) ^1^	27.4 ± 3.5	31.0 ± 3.5
Alanine aminotransferase (IU/L) ^2^	208.0 ± 45.2	231.0 ± 29.1
IL-4 (ng/mL) ^3^	3.1 ± 0.5	2.9 ± 0.3
IFN-γ (ng/mL) ^4^	4.3 ± 1.2	19.7 ± 1.4 *

Data are presented as the mean ± SEM or number. ^1^ 24 h, ^2^ 6 h, ^3^ 3 h, and ^4^ 12 h after alpha-galactosylceramide injection. * *p* < 0.01 (Student *t*-test).

**Table 2:** Role of natural killer T cells in mouse models of systemic lupus erythematosus.

Strain (Model), Age	Treatment	Outcomes	Ref.
NZB/NZW F1 (BWF1) mice	None	Expansion of NKT cells in association with the onset of the disease	[30]
MRL/lpr mice, 2 months of age	6 μg of alpha-galactosylceramide (α-GalCer) twice a week for 5 months	Improvement in inflammatory dermatitis without affecting renal disease	[34]
BALB/c and SJL mice (pristane-induced)	6 μg of α-GalCer twice a week for 1 month	Suppression of nephritis (BALB/c mice): exacerbation of nephritis (SJL mice)	[35]
BWF1 young mice	0.5 mg of anti-NK1.1 antibody three times a week for long periods	Amelioration of nephritis in late disease phase (worsening in early phase)	[37]
BWF1 mice, 20 weeks of age	4 μg of α-GalCer twice a week for 2 weeks	Enhancement of Th1 immune responses and exacerbation of nephritis	[38]
BWF1 mice, 7 weeks of age	4 μg of α-GalCer twice at a 3-day interval	Suppression of IL-10 production and reduction of severe proteinuria	[39]
BWF1 mice, 24 weeks of age	2 μg of α-GalCer once a week for 4 weeks	Suppression of Th2 immune responses and amelioration of nephritis	[41]
